# Changes in *SAM2* expression affect lactic acid tolerance and lactic acid production in *Saccharomyces cerevisiae*

**DOI:** 10.1186/s12934-014-0147-7

**Published:** 2014-10-30

**Authors:** Laura Dato, Nadia Maria Berterame, Maria Antonietta Ricci, Paola Paganoni, Luigi Palmieri, Danilo Porro, Paola Branduardi

**Affiliations:** Dipartimento di Biotecnologie e Bioscienze, Università degli Studi di Milano-Bicocca, Piazza della Scienza 2, 20126 Milan, Italy; Dipartimento di Bioscienze, Biotecnologie e Biofarmaceutica, Università degli Studi di Bari Aldo Moro, Via Orabona 4, 70125 Bari, Italy

**Keywords:** Lactic acid production, Lactic acid stress, *Saccharomyces cerevisiae*, S-Adenosylmethionine (SAM), *SAM2*

## Abstract

**Background:**

The great interest in the production of highly pure lactic acid enantiomers comes from the application of polylactic acid (PLA) for the production of biodegradable plastics. Yeasts can be considered as alternative cell factories to lactic acid bacteria for lactic acid production, despite not being natural producers, since they can better tolerate acidic environments. We have previously described metabolically engineered *Saccharomyces cerevisiae* strains producing high amounts of L-lactic acid (>60 g/L) at low pH. The high product concentration represents the major limiting step of the process, mainly because of its toxic effects. Therefore, our goal was the identification of novel targets for strain improvement possibly involved in the yeast response to lactic acid stress.

**Results:**

The enzyme *S*-adenosylmethionine (SAM) synthetase catalyses the only known reaction leading to the biosynthesis of SAM, an important cellular cofactor. SAM is involved in phospholipid biosynthesis and hence in membrane remodelling during acid stress. Since only the enzyme isoform 2 seems to be responsive to membrane related signals (*e.g.* myo-inositol), Sam2p was tagged with GFP to analyse its abundance and cellular localization under different stress conditions. Western blot analyses showed that lactic acid exposure correlates with an increase in protein levels. The *SAM2* gene was then overexpressed and deleted in laboratory strains. Remarkably, in the BY4741 strain its deletion conferred higher resistance to lactic acid, while its overexpression was detrimental. Therefore, *SAM2* was deleted in a strain previously engineered and evolved for industrial lactic acid production and tolerance, resulting in higher production.

**Conclusions:**

Here we demonstrated that the modulation of *SAM2* can have different outcomes, from clear effects to no significant phenotypic responses, upon lactic acid stress in different genetic backgrounds, and that at least in one genetic background *SAM2* deletion led to an industrially relevant increase in lactic acid production. Further work is needed to elucidate the molecular basis of these observations, which underline once more that strain robustness relies on complex cellular mechanisms, involving regulatory genes and proteins. Our data confirm cofactor engineering as an important tool for cell factory improvement.

**Electronic supplementary material:**

The online version of this article (doi:10.1186/s12934-014-0147-7) contains supplementary material, which is available to authorized users.

## Background

Lactic acid and its production by lactic acid bacteria (LAB) have a long history in the food industry for its application as an acidulant, flavouring agent, pH buffering agent, or preservative [1-4]. Microbial processes for its production have been established early in the last century. However, the commercial production of the purified acid in large-scale by microorganisms is relatively new. The production and applications of its derivative polylactic acid (PLA) [5,6] currently elicited an increased interest in optically pure lactic acid. Furthermore, the presence of both carboxylic and hydroxyl groups in the lactic acid molecule enables its conversion into different technologically useful chemicals such as pyruvic acid, acrylic acid, 1,2-propanediol and lactate ester via chemical and biotechnological routes [2,3,7,8], making it a primary chemical platform.

Initially, the natural producers were the “bio-catalysts” of choice for industrial lactic acid fermentations [9,10]. However, LAB require complex nutrients and are inhibited by the product, especially at low pH. The most relevant bottleneck in production by LAB is in all likelihood related to the inhibitory effects of the low pH of the medium on cell growth, cell viability and in turn on lactic acid accumulation. Indeed, large amounts of CaCO_3_ must be added during fermentation, to maintain a constant pH of the culture broth (at around 5) and sustain production. Under these conditions the final product is lactate, since the pK_a_ of lactic acid is 3.86. This in turn increases the operation costs for separation and purification of the desired product, which is actually the free acidic form [2,3,11], and therefore the acidification of the spent medium at the end of the fermentation becomes a required step.

The use of naturally low-pH tolerant organisms, such as yeasts, represents an alternative production route. In 1994 Dequin and Barre [12] first described a metabolically engineered *Saccharomyces cerevisiae* strain expressing a heterologous L-lactate dehydrogenase, obtaining a hetero-fermentative strain producing both ethanol and lactic acid. Since then, many improvements have been obtained along the years. Among them, (*i*) the deletion of pyruvate decarboxylase gene (s) to avoid ethanol production and increase production, productivity and yield of lactic acid [13-15], (*ii*) the increased yields due to the effect of different *S. cerevisiae* backgrounds and heterologous L-lactate dehydrogenases [16], (*iii*) the development of high-producing strains following classical selection methods, by direct exposure of the cells to the stressor, and indirect screenings by sorting the cells on the basis of tolerance-related traits like the capability to keep an higher intracellular pH [17,18], and (*iv*) the effect of overexpression of the hexose transporters (*e.g.* Hxt1p and Hxt7p) on glucose uptake and lactic acid productivity and production [19]. Metabolically engineered *S. cerevisiae* strains were also characterized for their energetic balance, showing that lactate production does not contribute to the net ATP production probably due to energy utilization for lactate export [20]. Recently, metabolically engineered yeast came on the market for lactic acid production (NatureWorks®) [21].

In spite of their ability to produce high levels of lactic acid at low pH, the presence of the undissociated weak acid in the growth medium imposes a high degree of stress to yeast cells [22-26]. The cell membrane is, in fact, selectively permeable to small polar and to hydrophobic molecules, like undissociated weak organic acids, which can cross it by passive diffusion following their gradient [27]. Because of the relatively high intracellular pH value, weak acids dissociate once into the cytoplasm, releasing H^+^ and the corresponding anion. Accumulation of both species has detrimental effects on cells, ranging from lowering of intracellular pH and inhibition of metabolic activities, to interference with lipid organization and membrane permeability/functions and induction of oxidative stress and cell death (reviewed in [22,23]), among others. Therefore, during detoxification, the protons are expelled via the H^+^-ATPase pump and the anions via active export systems (or metabolized), consuming huge amounts of energy. There is no surprise then in finding that membrane lipids and proteins are among the first targets of modification induced by some specific stresses [28-32].

Stress responses induce a complex cellular reprogramming. Classically, most metabolic engineering studies have focused on enzyme levels and on the effect of the amplification, addition, or deletion of a particular pathway directly linked with the product of interest. However, the current status of metabolic engineering is still hindered by the lack of our full understanding of cellular metabolism. Indeed, the complex aspects of integrated dynamics and overall control structure are the common obstacles for the optimal design of pathways to achieve a desired goal. Since cofactors are essential to a large number of biochemical reactions, their manipulation is expected to have large effects on metabolic networks. It is conceivable that cofactor availability and the proportion of cofactor in the active form may be critical in dictating the overall process yield. It has already been shown that cofactors play a major role in the production of different fermentation products (see, as example [33]). Furthermore, changes in cofactor pools induce changes at the transcriptional level as well as at the enzyme levels [34].

SAM (or AdoMet) is a central coenzyme in the metabolism that participates to a very high number of reactions [35]. In particular it functions as a donor of methyl groups to proteins, lipids, nucleic acids, vitamin B12 and others by SAM-dependent methyltransferases; it is also a precursor molecule in the aminopropylation and transulfuration pathways [36] and it regulates the activities of various enzymes. SAM has a role in the modelling of the plasma membrane structure, since it donates three methyl groups during the synthesis of phosphatidylcholine (PC) from phosphatidylethanolamine (PE). Malakar *et al*. [37] demonstrated a protective role of externally added SAM in *S. cerevisiae* cells growing under inorganic acid (HCl) stress, which they associated to the measured increase in PC:PE ratio and to the higher activity of the proton pump Pma1p. Moreover, SAM displays an anti-apoptotic role, acting as an indirect scavenger of reactive oxygen species (ROS) via enhancement of glutathione biosynthesis [38].

We therefore focused our attention on SAM-synthetase which catalyses the only known reaction that, starting from L-methionine (Met) and ATP, leads to the biosynthesis of SAM [39-41]. Notably, *S. cerevisiae* has two distinct SAM-synthetase genes, named *SAM1* and *SAM2*, which arose from gene duplication [42,43] and share a high degree of similarity (83% identity in the ORF, 92% in the translated sequence) [43]. Although *SAM1* and *SAM2* have at least partially overlapping functions, their regulation is different. Both genes undergo feedback repression by SAM, like other genes of the sulfur aminoacids metabolism, but the expression of *SAM2* also increases during growth, in a Sam2p-dependent manner [44]. Remarkably, *SAM2* is repressed after the addition of myo-inositol and choline, suggesting that Sam2p, but not Sam1p, is involved in phospholipid biosynthesis [45]. It is very likely that Sam2p is concerned to this process also during lactic acid stress.

In this work, the expression and localization of Sam2p under lactic acid treatment were evaluated. To assess the role of this protein during lactic acid stress, *SAM2* was both overexpressed and deleted in *S. cerevisiae* laboratory strains. Moreover, when *SAM2* was deleted in the engineered and evolved lactic acid producing strain CEN.PK m850 [18], higher lactic acid productivity and production were obtained.

## Results

### Sam2p as a putative responsive element to lactic acid stress

Based on the reported beneficial effects of SAM during inorganic acid (HCl) stress [37] and its involvement in membrane remodelling, we evaluated the protein levels of Sam2p by western blot analysis during lactic acid exposure. A chromosomal tagging approach by which the GFP coding sequence was fused in frame to the C-terminal coding region of the endogenous copy of the *SAM2* gene has been applied (see Methods). The *SAM2*GFP strain was created in the CEN.PK 113-11C background, a robust *S. cerevisiae* reference strain, and also in the BY4741 background, commonly used for functional genetic studies (EUROSCARF collection http://web.uni-frankfurt.de/fb15/mikro/euroscarf/).

The BY4741 *SAM2*GFP and CEN.PK *SAM2*GFP strains were grown in minimal medium with 2% w/v glucose in the absence and in the presence of different concentrations of lactic acid (pH 5, pH 3, 12 g/L and 20 g/L lactic acid at pH 3) and Sam2p levels were estimated using an anti-GFP antibody at 16 and 40 hours after inoculation, respectively corresponding to the exponential and the early stationary phase of growth. The biomass accumulation and the growth phase among the different conditions within the same genetic background were similar, for each time point considered. As control, β-actin levels were also detected.

Two analyses were run in parallel: in the first, the total protein fraction was extracted with trichloroacetic acid (TCA); in the second, three sub-fractions resulting from sequential protein extraction were separated: the first containing only soluble proteins, the second containing insoluble proteins solubilized with urea, the third containing highly insoluble proteins excluded from the second fraction and solubilized with concentrated sodium dodecyl sulfate (SDS) (see Methods for details).

**Figure 1:**
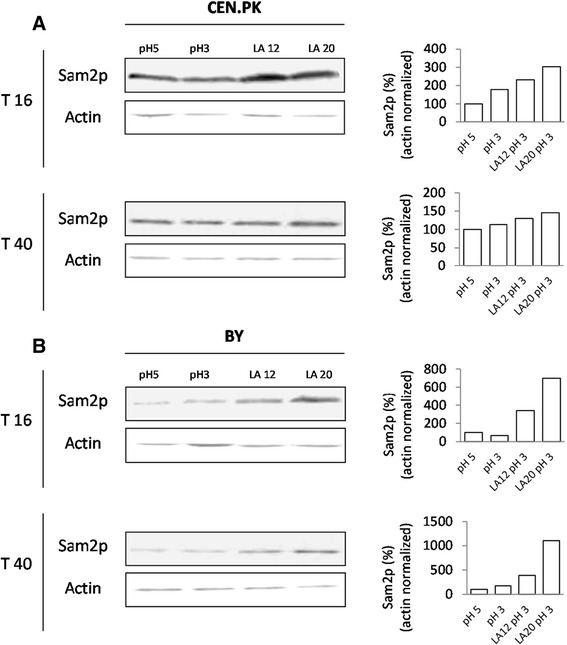
**Western blot analysis of total Sam2p levels in cells grown in the absence and presence of lactic acid.** CEN.PK 113-11C **(panel A)** and BY4741 *SAM2*GFP **(panel B)** cells were grown in shake flasks in minimal (YNB) medium with 2% w/v glucose without or with the addition of different concentrations of lactic acid (pH 5, pH 3, 12 g/L and 20 g/L lactic acid at pH3) and the Sam2p-GFP levels were evaluated after 16 and 40 hours after inoculation in the total protein fraction, extracted with TCA, using an anti-GFP antibody. Samples were normalised according to cell number. β-actin levels have been detected as control. Bands have been quantified by ImageJ 1.48 software. Histograms refer to the ratio (%) of Sam2p/Actin normalized to the values at pH 5. LA: lactic acid.

Figure 1 shows the western blots of the TCA extracts for CEN.PK (panel A) and BY (panel B) strains. Remarkably, in both strains the signal intensity of Sam2p-GFP increased in the presence of lactic acid, particularly in the BY strain (see panels B).

Noteworthy, Sam2p was found in all the three protein sub-fractions after sequential extraction. Additional file 1: Figure S1 shows the western blots obtained for CEN.PK. At 16 h, the signals detected in the soluble protein fractions were rather similar among the different conditions, thus the protein increase of the lactic acid samples was mainly ascribable to the highly insoluble protein fractions and to a lesser extent to the fractions solubilised with urea. This was also true for lactic acid samples collected at 40 h, when slight enrichments were also found in the native extracts (data not shown).

Overall, lactic acid determined an increase in the total amount of the Sam2p in both yeast backgrounds.

### Sam2p localization under lactic acid exposure

The localization of Sam2p is still a matter of debate. In fact, while the *LoQate* database [46] and Tkach *et al.* [47] report a cytoplasmic localization, the *Yeast GFP Fusion Database* [48] reports it as ambiguous, the *OrganelleDB* (A. Kumar’s Lab, Life Sciences Institute, University of Michigan; http://organelledb.lsi.umich.edu/) reports it as unknown and finally the *YPL*^*+*^*Database* (Oskolkova, Leitner and Kohlwein, personal communication) describes it as nuclear. Based on our previous data, therefore, the possible effects of lactic acid exposure on Sam2p-GFP fusion protein localization in the BY4741 *SAM2*GFP and CEN.PK *SAM2*GFP were investigated by fluorescence microscopy.

**Figure 2:**
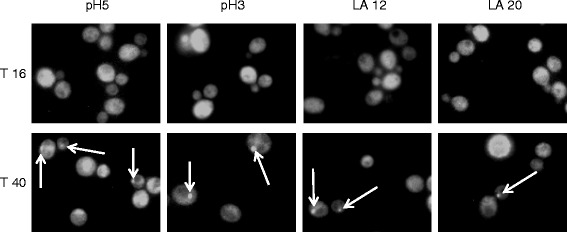
**Sam2p-GFP fluorescence distribution during growth in the absence and presence of lactic acid.** CEN.PK 113-11C *SAM2*GFP cells were grown in shake flasks in minimal (YNB) medium with 2% w/v glucose without or with the addition of different concentrations of lactic acid (pH 5, pH 3, 12 g/L and 20 g/L lactic acid at pH3). Epifocal microscope images were taken at 16 and 40 hours after inoculation, corresponding to exponential growth phase and early stationary phase, respectively. Pictures show Sam2p-GFP fluorescence in the green field. White arrows indicate Sam2p-GFP *foci*. LA: lactic acid.

Yeast cells were grown in the same conditions described above and observed under epifocal microscope at 16 and 40 hours after inoculation. The images of Figure 2, depicting CEN.PK cells, show that the presence of lactic acid had no significant effects on Sam2p-GFP distribution. At 16 h (upper panels) the signal was diffused into the whole cell, with the exclusion of extended dark areas representing the vacuoles and nuclei (based on DAPI staining, not shown). Therefore, the localization appeared to be mainly cytoplasmatic, although a contingent association with membranes cannot be excluded. At 40 h (bottom panels), instead, discrete spots emerging from the diffused fluorescence signal were visible. A similar situation was observed in BY4741 cells (data not shown). The number and dimensions of these *foci* were highly variable in all cells, irrespective of whether lactic acid was present or not. Therefore the data reported do not allow additional speculations on their relevance to stress tolerance. The nature of the observed Sam2p *foci*, never reported in literature before, is still unknown, and its biological significance needs to be further investigated.

In conclusion, Sam2p distribution within the cytosol in both yeast strains appeared to change in correlation with the growth phase.

### Effect of *SAM2* overexpression and deletion on lactic acid tolerance

The differential accumulation of Sam2p observed by the western blot analysis opens the question about a possible role of this protein during the cellular response to lactic acid stress at low pH. Consequently, the effect of *SAM2* overexpression was examined for growing cells challenged with different concentrations of the stressing agent. The wild type CEN.PK 102-3A and BY4741 strains were transformed with the pTEF-L-SAM2 multicopy plasmid (see Methods), carrying *SAM2* under the control of the strong constitutive *S. cerevisiae* TEF1 promoter. CEN.PK 102-3A and BY4741 cells transformed with the respective empty plasmid were used as controls.

**Figure 3:**
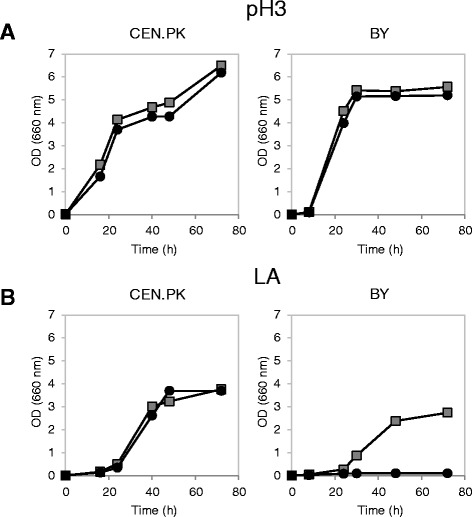
**Growth of wild type and** ***SAM2*** **overexpressing cells in the absence and presence of lactic acid.** Yeast cells were grown in shake flasks in minimal (YNB) medium with 2% w/v glucose at initial pH 3, without **(panel A)** or with **(panel B)** 40 g/l of lactic acid. Growth was determined as OD at 660 nm. Left panels: CEN.PK cells; right panels: BY cells. Dark grey squares: wild type control strains (CEN.PK 102-3A [pTEF-L], BY4741 [pTEF-L]). Black circles: cells overexpressing *SAM2* (CEN.PK 102-3A [pTEF-L-SAM2], BY4741 [pTEF-L-SAM2]).

Figure 3 shows the results obtained by cultivation in minimal medium with 2% w/v glucose without or with lactic acid (40 g/L) at pH 3. No remarkable differences were observed between the control and the *SAM2* overexpressing strains during growth without lactic acid at low pH, in both yeast backgrounds (Figure 3A). Lactic acid had a clear negative effect on the growth of all strains, visible in terms of growth delay and lower biomass accumulation (Figure 3B). However, while wild type and *SAM2* overexpressing cells grew similarly in the stressed condition for the CEN.PK background, in the BY background a marked difference between the two strains was observed, where surprisingly the *SAM2* overexpressing strain was much more affected compared to the control.

Since unexpectedly *SAM2* gene overexpression did not help improving lactic acid tolerance in the CEN.PK background and caused severe growth deficiencies in the BY4741 background, the effect of its deletion was also tested. *SAM2* was deleted in the CEN.PK 102-3A and BY4741 parental strains and in the same strains harbouring the pTEF-L plasmid (the backbone plasmid used for *SAM2* overexpression), complementing the leucine auxotrophy, to allow a direct comparison of all the data.

**Figure 4:**
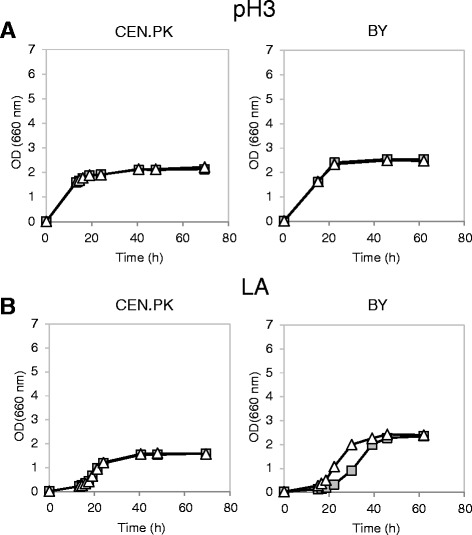
**Growth of wild type and** ***sam2Δ*** **leucine nutritionally complemented strains in the absence and presence of lactic acid.** Yeast cells were grown in shake flasks in minimal (YNB) medium with 2% w/v glucose and 50 mg/L of the necessary nutritional supplements at initial pH 3, without **(panel A)** or with **(panel B)** 34 and 38 g/l of lactic acid for CEN.PK (left panels) and BY (right panels), respectively. Growth was determined as OD at 660 nm. Light grey squares: parental wild type strains (CEN.PK 102-3A, BY4741). White triangles: *sam2Δ* cells (CEN.PK 102-3A *sam2Δ*, BY4741 *sam2Δ*).

**Figure 5:**
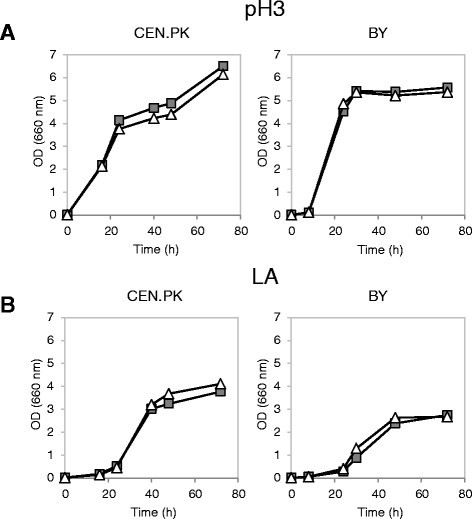
**Growth of wild type and** ***sam2Δ*** ***LEU2*** **genetically complemented strains in the absence and presence of lactic acid.** Yeast cells were grown in shake flasks in minimal (YNB) medium with 2% w/v glucose and 50 mg/L of the necessary nutritional supplements at initial pH 3, without **(panel A)** or with **(panel B)** 40 g/l of lactic acid. Growth was determined as OD at 660 nm. Left panels: CEN.PK cells; right panels: BY cells. Dark grey squares: wild type control strains (CEN.PK 102-3A [pTEF-L], BY4741 [pTEF-L]). White triangles: *sam2Δ* cells (CEN.PK 102-3A *sam2Δ* [pTEF-L], BY4741 *sam2Δ* [pTEF-L]).

Figures 4 and 5 show the growth curves obtained, respectively for the parental strains and for the LEU^+^ complemented strains. *SAM2* deletion had no effect, in all the tested strains, during growth in minimal medium at low pH (Figures 4A and 5A). When cells were stressed with lactic acid, once more no marked differences were observed in the CEN.PK background between the wild type and the deleted strain (Figures 4B and 5B). Interestingly, the BY4741 parental strain *sam2Δ* turned out to be less sensitive to the stressing agent than the wild type (Figure 4B): the specific growth rate in exponential phase was in fact 45% higher compared to control cells (0.11 ± 0.01 h^−1^*vs* 0.16 ± 0.01 h^−1^, mean and SD from three independent experiments). However, the complementation of leucine auxotrophy made void the positive impact of *SAM2* deletion on cellular growth (Figure 5B).

**Figure 6:**
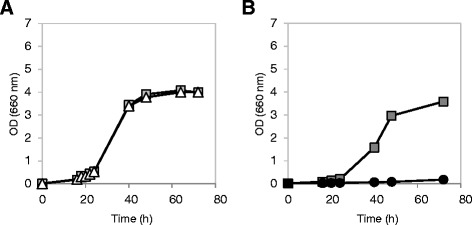
**Growth of wild type, deleted or overexpressing** ***SAM2*** **BY strains in minimal supplemented medium with lactic acid.** BY cells were grown in shake flasks in minimal (YNB) medium with 2% w/v glucose, 125 mg/L, 500 mg/L, 100 mg/L, 150 mg/L for histidine, leucine, methionine and uracil respectively, at initial pH 3, with 38 g/l of lactic acid. Growth was determined as OD at 660 nm. **Panel A**. Light grey squares: wild type control strain (BY4741) White triangles: *sam2Δ* cells (BY4741 *sam2Δ*). **Panel B**. Dark grey squares: wild type control strain (BY4741 [pTEF-L]) Black circles: cells overespressing *SAM2* (BY4741 [pTEF-L-SAM2]).

It has to be noticed that the final OD reached by the *leu*^*−*^ strains in the unstressed condition was lower compared to the values registered for LEU^+^ complemented strains, possibly indicating that the standard amino acid supplementation (50 mg/L) was not sufficient in the case of leucine. This effect was stronger in the CEN.PK background (Figure 4A). Pronk [49] suggested complementation of the medium with 125 mg/L, 500 mg/L, 100 mg/L, 150 mg/L for histidine, leucine, methionine and uracil respectively. Accordingly, the growth experiments, in which the effect of *SAM2* modulation has been observed, were repeated in the presence of lactic acid at pH 3 with the supplemented relevant chemicals (Figure 6). While in this medium *SAM2* deletion did not affect the cellular growth in the presence of the stressing agent (panel A), *SAM2* overexpression was still detrimental to the cells (panel B). This confirms that Sam2p recombinant overproduction is not beneficial to improve the tolerance to this stress.

### Effect of lactic acid pulsed stress on cell viability

**Figure 7:**
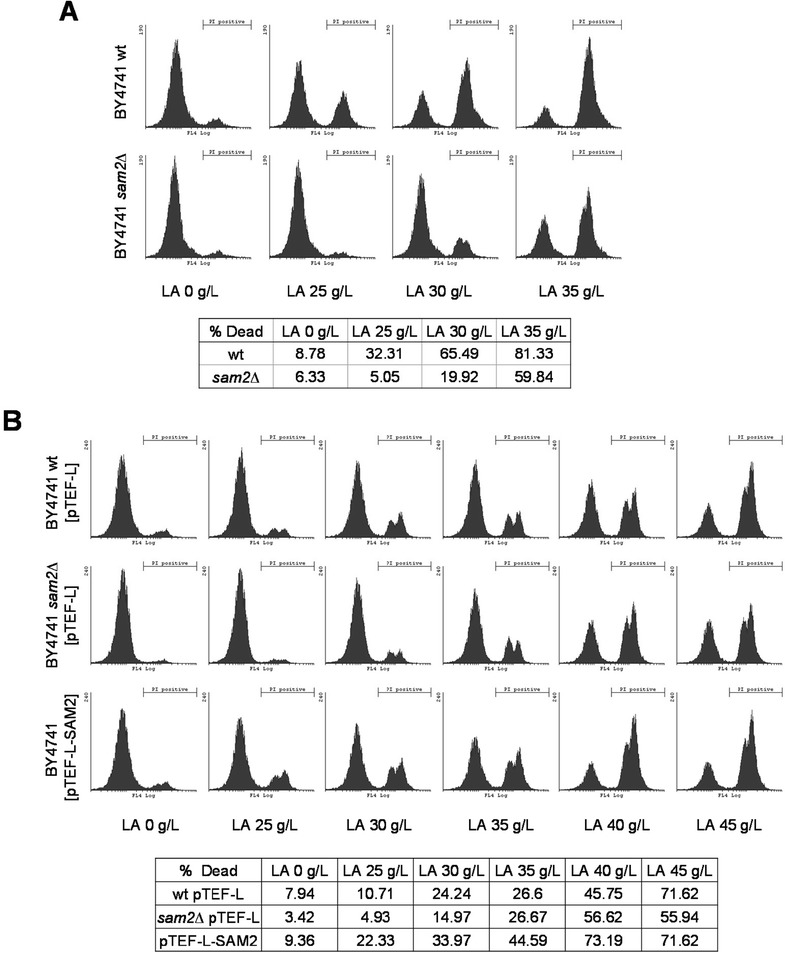
**Viability determination for cells stressed with lactic lacid.** Cells were grown in minimal medium until the exponential phase and then treated with a pulse of lactic acid. After 30 min of incubation, cells were collected and stained with propidium iodide (PI) to detect dead and/or severely damaged cells by flow cytometry. The fluorescence emission was measured through a 670 nm long pass filter (FL3 parameter). For each sample, 25000 cells were analysed. The bar indicates the PI positive subpopulation. **Panel A**: BY4741 and BY4741 *sam2Δ*. **Panel B**: BY4741 [pTEF-L], BY4741 *sam2Δ* [pTEF-L] and BY4741 [pTEF-L-SAM2].

The effect of *SAM2* deletion and overexpression was also evaluated in terms of cellular viability in the aforementioned strains, *i.e.* CEN.PK 102-3A and BY4741 wt, *SAM2* overexpressing and *sam2Δ* (complemented or not for leucine auxotrophy). Cells were grown in minimal medium, until the exponential phase was reached, and then treated with a pulse of lactic acid at different concentrations (0, 25, 30, 35, 40 and 45 g/L at pH 3). After 30 minutes the cells were collected, stained with propidium iodide (PI) and analyzed by flow cytometry to identify dead and/or severely compromised cells. Figure 7 shows the histograms obtained for the BY4741 strains, where the left peak corresponds to intact (PI-negative) cells, while the right peak corresponds to the dead/damaged (PI-positive) cells (we currently do not have an interpretation for the bimodal distribution visible in the plots).

As for the growth experiments, also in this case the effects of *SAM2* gene modulation were observed only in the BY background. In particular, the parental *leu*^−^ strain *sam2Δ* showed a percentage of dead/damaged cells consistently lower than the control strain (Figure 7A). When the leucine auxotrophy was complemented, however, the differences between the two strains were not significant (Figure 7B). On the contrary, and in agreement with what already observed in the kinetics of growth, the *SAM2* overexpressing strain showed an increased sensitivity to lactic acid stress, with a higher percentage of dead/damaged cells compared to the control (Figure 7B). It is worth to notice that for any tested lactic acid concentrations the BY *leu*^−^ strains had a higher mortality compared to LEU^+^ strains.

In the CEN.PK background, instead, the *SAM2* deletion and overexpression had no significant effect on cellular viability (data not shown).

### Analysis of intracellular AXP levels

**Figure 8:**
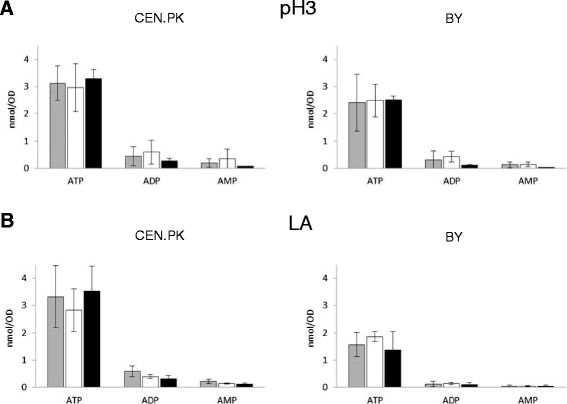
**Intracellular adenine nucleotides concentrations in the absence and presence of lactic acid.** Yeast cells were grown in shake flasks in minimal (YNB) medium with 2% w/v glucose at initial pH 3, without **(panel A)** or with **(panel B)** 40 g/l of lactic acid. ATP, ADP and AMP were extracted from samples collected during the exponential growth phase and determined by HPLC. Concentrations are expressed per OD of cell culture. The mean and SD for two independent experiments is reported. Left panels: CEN.PK cells; right panels: BY cells; Grey bars: CEN.PK 102-3A [pTEF-L], BY4741 [pTEF-L]; white bars: CEN.PK 102-3A *sam2Δ* [pTEF-L], BY4741 *sam2Δ* [pTEF-L]; black bars: CEN.PK [pTEF-L-SAM2], BY4741 [pTEF-L-SAM2].

Our data indicate that the Sam2 protein levels respond to lactic acid in both the CEN.PK and BY4741 yeast strains, but the effects of *SAM2* gene deletion and overexpression, at least in terms of growth and cell viability, are only detectable in the BY background. We considered the hypothesis that these differences might be correlated with different AXP pool composition. We therefore measured the adenine nucleotide content of CEN.PK 113-11C and BY4741 wt, *SAM2* overexpressing and *SAM2* deleted strains, complemented for leucine auxotrophy, during the exponential growth phase on minimal medium with 2% w/v glucose without or with lactic acid (samples were collected at OD ~1 if without and at OD ~0.3 if with lactic acid, respectively). The ATP, ADP and AMP (collectively referred as AXP) intracellular concentrations were determined by HPLC with the method from Ask *et al*. [50], as described in the Methods section. Data are reported in Figure 8, normalized for culture OD for consistency with the other data.

In the CEN.PK background (Figure 8A) similar levels of all the nucleotides were found in all the strains regardless the presence of lactic acid, and no differences were evident depending on *SAM2* expression levels. In the BY background, lower mean ATP levels were registered in the presence of lactic acid compared to control medium (Figure 8B), although again no specific differences were assessed in dependence on *SAM2* expression. Interestingly, a comparison of the data obtained for the two yeast backgrounds shows a lower mean ATP content in the BY strains compared to CEN.PK. The differences are statistically significant, with a Student’s *t*-test *p*-value of 0.012 for the comparison at pH 3 and of 0.004 for the comparison in lactic acid at pH 3. Also the ADP and AMP mean concentrations were lower in the BY strain, especially in the presence of lactic acid, so that the calculated energy charge resulted conserved in all the strains, at physiological levels higher than 0.8 (data not shown).

### Effect of *SAM2* deletion on lactic acid production by a *S. cerevisiae* strain engineered and evolved for the industrial process

Despite the fact that the mechanisms involved remain far from being elucidated, our data indicate that *SAM2* deletion might confer an advantage to cells exposed to lactic acid stress when the overall conditions are not optimal. Since the final goal of our studies is to find conditions that can bring advantages to lactic acid production, we tested the effects of *SAM2* deletion in the lactic acid producing strain during the production process. Indeed, even though the productive strain was originally derived from the robust CEN.PK background and does not bring any auxotrophies, still the production process puts it under extremely severe stress conditions.

The recombinant CEN.PK m850 strain is a homolactic fermenting cell factory able to produce up to 60 g/L in 60 h at pH values lower than 3. It was derived from the CEN.PK background via engineering steps that deleted all the pyruvate decarboxylase (*PDC*) genes and introduced the *L. plantarum* lactate dehydrogenase (LDH) activity, eliminating in this way all ethanol production in favour of lactic acid production from the free pyruvate. It furthermore underwent selection, following an adaptive laboratory evolution approach, for improved acid tolerance [18].

**Figure 9:**
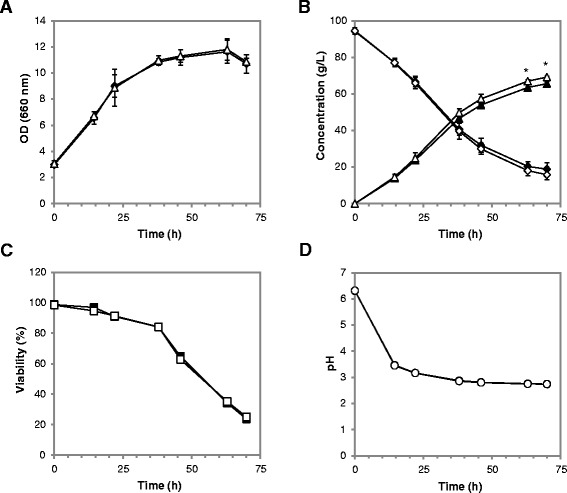
**Batch lactic acid production in wt and** ***sam2Δ*** **strains.** Fermentation profiles for CEN.PK m850 (filled symbols) and CEN.PK m850 *sam2Δ* (open symbols) pre-grown in shake flasks and then transferred in new flasks in minimal medium containing 90 g/L of glucose for the lactic acid production phase. **(A)** Biomass formation (OD at 660 nm). **(B)** Residual glucose (diamonds) and produced lactic acid (triangles). **(C)** Cellular viability was determined by PI staining followed by flow cytometry. **(D)** Culture medium pH. **Panel A** and **B** report the mean and SD for three independent experiments (*p ≤ 0.01; Student’s *t*-test; for supplementary details see Additional file 2: Table S1); **Panel C** and **D** report data from a single representative experiment.

The *SAM2* gene was deleted in the CEN.PK m850 strain, and the performances of the parental and the *sam2Δ* strains were compared during the production of lactic acid in minimal medium in the presence of high amounts of initial glucose. Cells were first pre-cultivated for 24 hours in minimal medium with 10 g/L ethanol and 0.5 g/L glucose, to obtain the biomass, and then transferred to a fresh medium containing 5 g/L ethanol and 90 g/L glucose for the production phase (as previously described, [17]). Figure 9 reports the culture parameters monitored at time intervals throughout the production phase: cellular growth (panel A), residual glucose and produced lactic acid in the medium, measured by HPLC (panel B), cell viability as determined by flow cytometry (panel C) and culture medium pH (panel D).

No differences (*p* > 0.05 Student’s *t*-test) were observed between the two strains in terms of biomass accumulation (Figure 9A) and cell viability, the latter assessed after staining with either PI (Figure 9C) or fluorescein diacetate (whose signal is linked to metabolically active cells; data not shown), and extracellular pH values were almost identical (Figure 9D). Instead, differences were measured for the glucose and lactic acid concentrations (Figure 9B), indicating higher specific lactic acid production rates for the CEN.PK m850 *sam2∆* strain compared to the control.

A mean 5.4% increase in lactic acid production was observed in the *sam2∆* strain at the end of the process (69.2 ± 0.6 *vs* 65.6 ± 0.9 g/L, average and SD of three independent experiments). Based on a two-tails, unpaired, heteroscedastic Student’s *t-*test, the differences in production at the last two time points of the experiment are highly significant (*p*-values 0.0103 and 0.0087 respectively at 63 and 70 h). The 95% confidence intervals (CI) for lactic acid production throughout the process were also calculated (Additional file 2: Table S1), indicating statistical significance for the differences found from the 46 h time point onward. For both strains, the yields were similar (0.88 ± 0.01 and 0.87 ± 0.03 g of lactic acid per g of glucose consumed, respectively for the *sam2∆* and the control strain). The differences observed between the two strains might be judged as small, but it must be considered that the cells were already pushed close to the theoretical limits (in terms of lactic acid yield) and in extreme conditions, therefore improvements of a high percentage cannot be expected.

**Figure 10:**
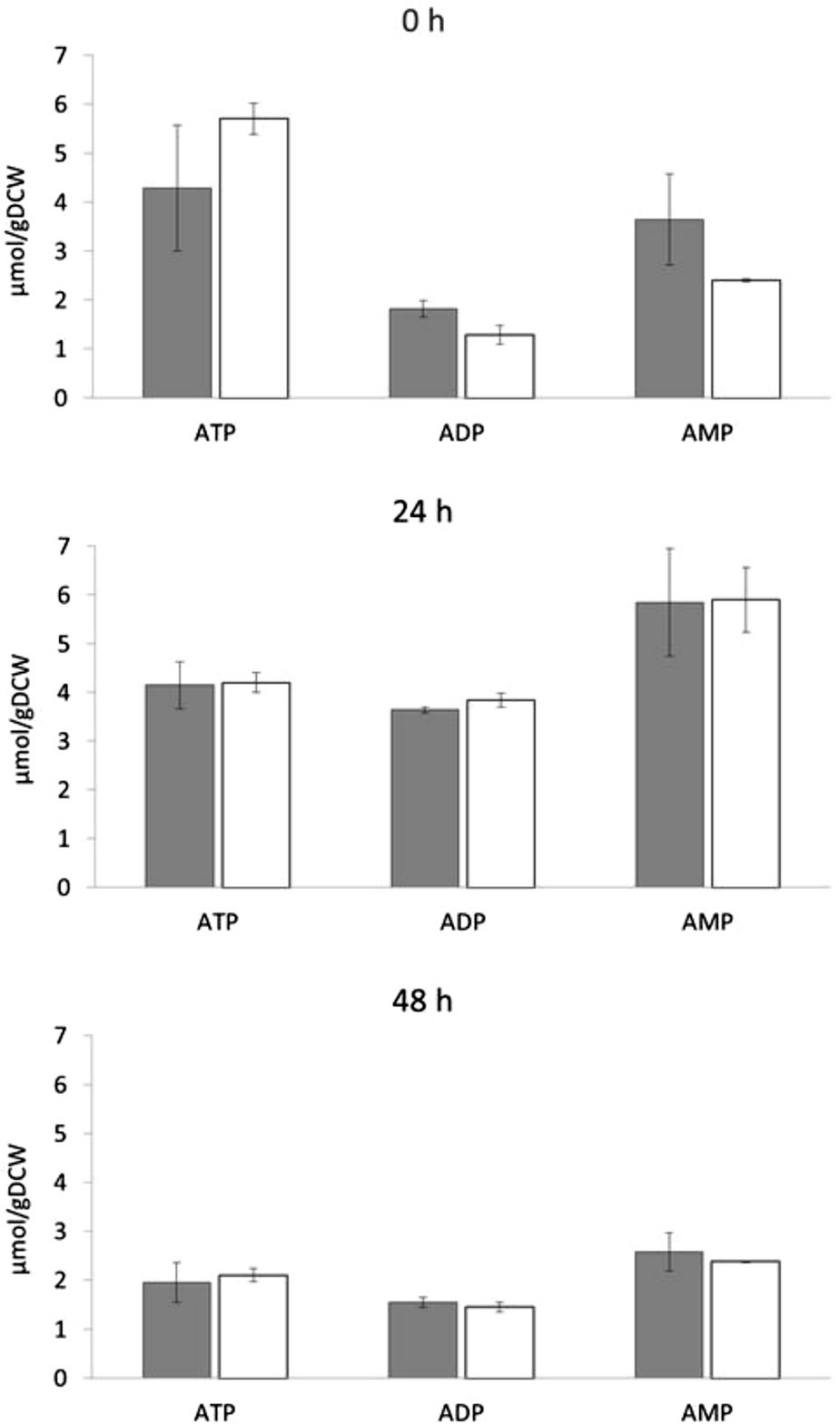
**Intracellular adenine nucleotides concentrations in the lactic acid producing strains.** CEN.PK m850 (grey bars) and CEN.PK m850 *sam2Δ* (white bars) were pre-grown in shake flasks and then transferred in new flasks in minimal medium containing 90 g/L of glucose for the lactic acid production phase. ATP, ADP and AMP were extracted and determined by HPLC, immediately before (0 h) or after the transfer in the production medium, at the indicated times (24 h and 48 h). Nucleotide concentrations are expressed per gram of dry cell weight (DCW). The mean and SD for two independent experiments is reported.

To test if energetic balance might contribute to the observed differences, the intracellular AXP concentrations were determined in the control and *sam2∆* strain during the process already described. Figure 10 shows the mean data and SD relative to cells analyzed immediately before inoculation (indicated as 0 h) and at 24 and 48 hours after the beginning of the production phase, respectively, in two independent experiments. At time 0 h, the ADP and AMP contents were lower whereas the ATP content was higher in the *sam2∆* strain compared to the control, despite a high variability in the case of ATP. After inoculation, no differences were found between the two strains. At 24 h, the ADP and AMP concentrations increased in both strains compared to 0 h, while at 48 h all the three species decreased.

Accordingly, the calculated energy charge (Table 1) was higher in the *sam2∆* strain immediately before the production phase (0 h), while there was no difference between the two strains later during production. Noteworthy, at all the time points and more pronouncedly during the production phase, the energy charge was below the physiological levels, differently from the laboratory strains, confirming the high stress experienced by the lactic acid producing strain.

**Table 1:** **Energy charge values in the lactic acid producing strains**

**Time (h)**	**m850**		**m850** ***sam2Δ***	
**0**	0.53	±0.11	0.68	±0.01
**24**	0.44	±0.02	0.44	±0.03
**48**	0.45	±0.07	0.48	±0.01

The mean and SD for two independent experiments are reported.

## Discussion

### A hypothesis on the mechanism triggering Sam2p increase upon lactic acid stress

SAM has a role in the modelling of the plasma membrane structure, taking part to the PC synthesis starting from PE. Phospholipids represent a major portion of the dry weight of a cell, they are essential for many different cellular processes and their alteration leads to membrane dysfunction [51]. As well, they are reservoirs of secondary messengers, provide precursors for the synthesis of macromolecules, serve in the modification of membrane association, and function as molecular chaperons (reviewed in [52]). Since PC is the most abundant phospholipid species in yeast membranes, it is not surprising that the pathway responsible for SAM synthesis and the pathway responsible for PC synthesis are transcriptionally and metabolically coordinated. The key element of this coordination was shown to be the *SAM2* gene [53], since it is the only gene directly regulated by both Met4p and Opi1p transcriptional factors.

Moreover, PC is the major phospholipid species in the yeast mitochondrial membranes [54], and it is required for mitochondrial respiratory functions [55]. Therefore, we could speculate that a signal inducing the increase of Sam2p in response to the toxic effects of lactic acid (shown in the western blot analysis) might be triggered by the increased need of PC synthesis.

### An apparent inconsistency

We initially hypothesized that *SAM2* overexpression might have a positive effect on cellular fitness. The reported antioxidant properties of SAM in mammals as well as in yeast [38,56,57] would have supported this hypothesis. The results presented demonstrated that in the BY4741 strain this was not the case, and instead the deletion of *SAM2* had a positive effect. Such an unexpected outcome has been described before for different gene products: for example, the expression of the gene encoding for the cell wall mannoprotein Sed1p was induced by exposure of *S. cerevisiae* cells to lactic acid, but its deletion conferred more resistance to the same stressor [58]. Moreover, *SED1* deletion in combination with the deletion of three genes (*DSE2*, *SCW11*, *EAF3*) identified after a screening for lactic acid resistance resulted in enhancement of the resistant phenotype of the single deleted mutants [59]. These findings might support the role of *SAM2* as stress-mediator, similarly to other stress-induced genes.

### Localization, distribution and abundance of Sam2p

The protein Sam2p has a predicted globular structure, with no transmembrane regions or signal peptide [60], like its homolog Sam1p; it is therefore predicted to be soluble. Its cellular localization in this work was shown by fluorescence microscopy to be mainly cytoplasmic during exponential growth phase, and then to change during the early stationary phase, showing scattered (cytoplasmic) *foci*. This might also explain the decreased solubility. To test a possible interaction with other proteins in complex (es) eventually associated to the membranes, we analysed plasma membrane enriched fractions (PMEF) of the strain CEN.PK, finding a statistically non significant (i.e. present in two out of three replicates) enrichment of a spot corresponding to Sam2p (our unpublished results). This might reflect its association in (homo or hetero) protein complexes, and a co-sedimentation with plasma membrane proteins during the extraction protocols. Overall, the data suggest that Sam2p is probably relocated in a different way in response to diverse stimuli, presumably requiring its function in different and specific pathways, which need to be further investigated. Co-immunoprecipitation and co-localization experiments will be very helpful to shed some light on this aspect.

### The outcomes of manipulating *SAM2* expression

If the observed increase in Sam2p levels is a cellular mechanism triggered to cope with lactic acid stress, why does the deletion of the corresponding gene determine higher lactic acid production in the m850 background? Our data on AXP concentrations suggest that the energetic balance might have a role. In fact, the higher ATP concentration found in the CEN.PK m850 *sam2∆* strain before starting the lactic acid production phase, together with a higher energy charge value, might account for the superior performance of this strain compared to the CEN.PK m850 parental strain. Probably, a higher biosynthetic potential endows the cells with a larger pool of beneficial metabolites and/or better sustain the activity of energy-consuming detoxifying systems. As it was previously demonstrated that lactic acid production in engineered *S. cerevisiae* is limited by ATP availability [20], the fact that no ATP or energetic differences were detected later on during production is not surprising, since it is highly probable that in such a dynamic situation any ATP excess would be readily used by the cells. In the laboratory strains, the different auxotrophies might also contribute to the different ATP levels, due to the energetic cost of amino acid intake. The substantially lower percentages of dead cells in the genetically complemented BY clearly indicate that prototrophy gives a substantial advantage during lactic acid stress. Besides that, other mechanisms might as well be involved in the different outcomes of *SAM2* expression in the two laboratory strains. More specifically, in the case of the BY4741 background *SAM2* overexpression caused severe growth deficiencies and increase cell death even if no specific differences in ATP levels were assessed in dependence on *SAM2* modulation. Furthermore, the positive impact of *SAM2* deletion was not significant when the leucine auxotrophy was complemented. Notably, it has been demonstrated that the leucyl-tRNA synthetase (LeuRS) triggers TORC1 activation [61], therefore promoting cell growth. The resulting biosynthetic pathways stimulation might cover the positive effect of Sam2p absence postulated in our hypothesis and might also account for cellular growth in more severe stress conditions (40 g/l of lactic acid). Despite more experimental evidences are necessary before further speculations can be proposed, the differences between/among genetic backgrounds are possibly ascribable to other pathways in which SAM is involved. For example, SAM is also consumed in the synthesis of ergosterol, and CEN.PK was shown to have a different regulation of the ergosterol biosynthesis pathway and different ergosterol contents compared to S288c (the progenitor of BY, [62]). Our data to date seem to suggest that the connection between lactic acid stress and Sam2p function is interconnected with many specific pathways, and it is not only ascribable to energy availability or auxotrophic requirement.

## Conclusions

Cofactor engineering, *i.e.* the manipulation of cofactor levels, as exemplified by SAM in this work, in addition to providing means to study cellular metabolism has the potential to be used as an additional tool to achieve desired metabolic engineering goals and fits with current trends in systems biotechnology. Our findings confirm the potential of cofactor-engineering strategies for industrial application [63].

Summarizing, at least four are the most relevant observations deriving from the current work. First of all, (*i*) lactic acid addition at low pH determines an increase of Sam2p in the cell. This increase was mainly associated to the insoluble protein fraction. In parallel, the fluorescence microscopy data highlighted the presence of protein aggregates appearing in stationary phase cells (Figure 2), whose further investigation might lead to novel insights on the dynamics of Sam2p (and Sam1p) interactions with other partners for the accomplishment of specific functions. This work hence added useful information on the cellular distribution of an enzyme of high importance for cell metabolism, whose localization is still reported as ambiguous.

Then, (*ii*) the overexpression of *SAM2* reduces the fitness of the laboratory strain BY4741 during lactic acid stress, while it has no obvious effects on the intrinsically more stress resistant laboratory strain CEN.PK 113-5D. On the contrary, (*iii*) the deletion of *SAM2* confers a growth advantage and a higher viability to BY4741 cells under lactic acid stress in a leucine auxothrophic strain, while again it has no obvious effects on the strain CEN.PK 113-5D.

Finally (*iv*) the deletion of *SAM2* allows a better production (g/L) and productivity (g/L h) of lactic acid from a previously engineered and evolved yeast strain.

All together, these data indicate Sam2p as a responsive element to lactic acid stress and suggest its modulation for lactic acid production improvement. Clarifying the nature of Sam2p interactions with other cellular components and their role in response to lactic acid stress might lead, in the future, to even higher resistance properties and productions via engineering of other interactors.

## Methods

### Yeast strains, transformation, media and cultivation

The *S. cerevisiae* parental and derived strains used in this study are listed in Table 2. Strain CEN.PK 102-3A was used for overexpression/deletion studies and CEN.PK 113-11C for GFP fusion. BY4741 (obtained from EUROSCARF) was used for overexpression/deletion studies and GFP fusion. The m850 lactic strain has been previously described [17,18], obtained starting from a *PDC1*, *PDC5*, *PDC6* triple deleted CEN.PK strain [20], and was here deleted in *SAM2*.

**Table 2:** **Yeast strains used and created in this study**

**Strain**	**Relevant genotype**	**Plasmid**	**Reference**
CEN.PK 113-11C	*MATa, ura3-52, his3-*Δ*1*	-	P. Kotter^1^
CEN.PK 102-3A	*MATa, ura3-52, leu2-3,112*	-	P. Kotter^1^
CEN.PK [pTEF-L]	CEN.PK 102-3A	pTEF-L, multicopy	This work
		(*Sc*TEF1, *LEU2*)	
CEN.PK [pTEF-L-SAM2]	CEN.PK 102-3A	pTEF-L-SAM2	This work
		(*Sc*TEF1, *ScSAM2*, *LEU2*)	
CEN.PK *sam2Δ*	CEN.PK 102-3A *sam2::KanMX4*	-	This work
CEN.PK *sam2Δ* [pTEF-L]	CEN.PK 102-3A *sam2Δ*	pTEF-L	This work
		(*Sc*TEF1, *LEU2*)	
CEN.PK *SAM2*GFP	CEN.PK 113-11C	-	This work
	*SAM2:: SAM2*GFP-*HIS3*		
CEN.PK m850	*MATa, pdc1(-6,-2)::loxP,*	YEpLpLDH	[18]
	*pdc5(-6,-2)::loxP, pdc6 (-6,-2)::loxP, ura3-52, acid tolerant*	(*Sc*TPI, *Lp*LDH, *URA3*)	
CEN.PK m850 *sam2Δ*	CEN.PKm850 *sam2::KanMX4*	YEpLpLDH	This work
		(*Sc*TPI, *Lp*LDH, *URA3*)	
BY4741	*MATa, his3-*Δ*1, leu2-*Δ*0, met15-*Δ*0, ura3-*Δ*0*	-	EUROSCARF^2^
BY4741[pTEF-L]	BY4741	pTEF-L	This work
		(*Sc*TEF1, *LEU2*)	
BY4741[pTEF-L-SAM2]	BY4741	pTEF-L-SAM2	This work
		(*Sc*TEF1, *ScSAM2*, *LEU2*)	
BY4741 *sam2Δ*	BY4741 *sam2::kanMX4*	-	This work
BY4741 *sam2Δ* [pTEF-L]	BY4741 *sam2Δ*	pTEF-L	This work
		(*Sc*TEF1, *LEU2*)	
BY4741 *SAM2*GFP	BY4741 *SAM2:: SAM2*GFP-*HIS3*	-	This work

^1^Institut fur Mikrobiologie der Johann Wolfgang Goethe Universitat, Frankfurt, Germany.

^2^ http://web.uni-frankfurt.de/fb15/mikro/euroscarf/.

Yeast transformations were performed according to the LiAc/PEG/ss-DNA protocol [64] and the strains were transformed with the constructs described below, in parallel with the corresponding empty plasmids. Integration of the constructs was confirmed by PCR analysis. For each set of transformation at least three independent transformants were initially tested, showing no significant differences among them.

Yeast cultures were performed in synthetic minimal medium (0.67% w/v YNB Biolife without amino acids) with 2% w/v D-glucose as carbon source. When required, supplements such as leucine, uracil, methionine and histidine were added to a final concentration of 50 mg/L, or to 125 mg/L, 500 mg/L, 100 mg/L, 150 mg/L for histidine, leucine, methionine and uracil respectively for the experiment shown in Figure 6, while the antibiotic G418 (Roche Diagnostics) was added to a final concentration of 200 mg/L. Lactic acidic stress was imposed by adding the desired amount of L-lactic acid (Sigma-Aldrich) to the culture medium. The final media have been prepared starting from 2 different stock solutions, one of 100 g/L lactic acid and one of synthetic minimal medium 2X, in order to obtain the desired lactic acid concentration. The pH of the lactic acid and the culture media were adjusted to 3 with pellets of KOH and HCl 1 M, respectively. Cell growth was monitored by measuring the OD at 660 nm at regular time intervals and cells were inoculated at an initial OD of 0.02 for growth kinetics experiments and at an initial OD of 0.005 for western blot and fluorescence microscopy experiments. All cultures were incubated in shake flasks at 30°C and 160 r.p.m. and the ratio of flask/medium volume was 5/1.

For the lactic acid pulsed stress experiment, aliquots of exponentially growing cultures were transferred in tubes containing the desired amount of lactic acid, adjusted to pH 3, at a final OD of 0.1. The cells were incubated at 30°C and 160 r.p.m. for 30 min.

The producing strain CEN.PK m850 and the derived transformants were cultivated as previously described [17]. Briefly, after a first batch growth phase, cells were collected by centrifugation and resuspended in fresh medium at a final OD of 3; lactic acid production kinetics were then performed by incubating at 32°C and 185 r.p.m. in 250-mL quadruple baffled shake flasks in minimal medium containing 2.78 g/L CaCO_3_, 1.7 g/L YNB without amino acids and without (NH4)_2_SO_4_, 1 g/L urea, 5 ml/L ethanol, and with different glucose concentration (70, 80, 90 g/L) as carbon source. Each experiment was repeated at least three times.

### Gene amplification and plasmids construction

The *S. cerevisiae SAM2* gene sequence was amplified by PCR using as a template the genomic DNA from CEN.PK strain, extracted by standard methods [65]. Pwo DNA polymerase (Roche catalogue no. 11 644 955 001) was used on a GeneAmp PCR System 9700 (PE Applied Biosystem, Inc.). Standard conditions used were 0.2 mM primers, 1.5 U of Pwo and 3 μL of genomic DNA. The program used for amplification of gene was as follows: after 5 min at 94°C, 30 cycles (each cycle consisting of 45 sec at 94°C, 30 sec at 58°C and 1 min 30 sec at 72°C) were carried out, followed by 7 min at 72°C. Oligonucleotides pairs for *SAM2* were as follows: SAM2_fw (5′-AATCATGTCCAAGAGCAAAACTTTCTTAT-3′) and SAM2_rev (5′-CATGGGAAAAACCAAAGAAATTGGAATTTTAA -3′). The amplified fragment was sub-cloned using the Perfectly Blunt Cloning kit (Novagen) into the *Escherichia coli* vector pSTBlue-1 obtaining the plasmid pSTBlue-SAM2. The insert was sequenced and it resulted identical to the deposited *S. cerevisiae* target sequence (*SAM2*, GeneID: 852113). This coding sequence was used for the construction of the multicopy expression plasmid pTEF-L-SAM2. This plasmid was derived from the commercial yeast multicopy expression plasmid p427-TEF (Dualsystems Biotechnology, CH), upon substitution of the selective marker Kan-MX with *LEU2* as follows: p427-TEF was *Nco*I digested, blunted and *Dra*III digested. The *LEU2* marker was excised from pYX042 (R & D Systems, Inc., Wiesbaden, D) by digestion with *Not*I, followed by blunting, and *Dra*III digestion, and then ligated to the recipient vector. The obtained vector pTEF-L was linearized with *Eco*RI and ligated to the *SAM2* ORF, excised with *Eco*RI from pSTBlue-SAM2.

The disruption of *SAM2* was performed using a standard recombination approach. pSTBlue-SAM2 was *Nco*I digested, blunted and *Eco*RV digested in the *SAM2* ORF. The excided fragment of about 200 nt was replaced with Kan-MX. The Kan marker was obtained from pFA6A-KanMX4 [66] digested with *Eco*RV and *Bam*HI. The deletion cassette *SAM2*sx-KanMX-*SAM2*dx was excised from the resulting plasmid by cutting with *Nde*I and *Pvu*II and used directly for yeast transformations. The obtained clones were screened by PCR using the following conditions: 5 min at 94°C, 30 cycles (45 sec at 94°C, 45 sec at 58°C and 2 min at 72°C) and 7 min at 72°C. The control primers, SAM2_fw_gen (5′-CGACGTCAAATCTTCATATGCAAGG-3′) and Kan_fw (5′-AACGTGAGTCTTTTCCTTACCCAT-3′), were designed upstream of the ATG and in the KanMX marker cassette. The DyNAzyme™ II DNA Polymerase (Finnzymes Reagents) was utilized for those reactions. DNA manipulation, transformation and cultivation of *E. coli* (Novablue, Novagen) were performed following standard protocols [65]. All the restriction and modification enzymes utilised are from NEB (New England Bio- labs, UK) or from Roche Diagnostics.

The substitution of *SAM2* endogenous ORF with the construct *SAM2*GFP was performed using a standard recombination approach. The construct was obtained by PCR using as template the Longtine plasmid pFA6a-GFP (S65T)-His3MX6. Standard conditions used were 0.2 mM primers, 1.5 U of Pwo and 0.3 μL of plasmid DNA. The program used for amplification of construct was as follows: 5 min at 94°C, 5 cycles (45 sec at 94°C, 30 sec at 50°C and 2 min at 72°C) and 7 min at 72°C, then 20 cycles (45 sec at 94°C, 30 sec at 65°C and 2 min at 72°C) and 7 min at 72°C. Oligonucleotides pairs for *SAM2*GFP were as follows: SAM2_Fw_longtine (5′-TCAAGAGTACTCATGGGAAAAACCAAAGAAATTGGAATTTCGGATCCCCGGGTTAATTAA-3′) and SAM2_Rev_longtine (5′-TATAAAAATCAAAATAAAACATTTATTGTCTAAATGTTTAGAATTCGAGCTCGTTTAAAC-3′). The amplified fragment was used directly for yeast transformation.

The obtained clones were screened by PCR using the following conditions: 5 min at 94°C, 30 cycles (45 sec at 94°C, 45 sec at 57.5°C and 1 min 30 sec at 72°C) and 7 min. the control primers were as follows: SAM2_fw_gen (5′-CGACGTCAAATCTTCATATGCAAGG-3′) and Gfp_Rev (5′-AAGAATTGGGACAACTCCAGTGA-3′). The DyNAzyme™ II DNA Polymerase (Finnzymes Reagents) was utilized for those reactions.

### Protein extractions for western blot analysis

#### Total protein extraction

10^8^ cells were broken by glass beads in 20% TCA. After centrifugation, the pellet was resuspended in the Laemmli buffer system and in 1 M Tris, pH 7. The sample was boiled for 3 min and after centrifugation the supernatant was collected for the western blot analysis.

#### Tris-Urea-SDS sequential extraction

10^8^ cells were resuspended in Tris buffer (50 mM Tris pH 8.7, 150 mM NaCl, 1 mM EDTA, 1 mM protease inhibitor cocktail, 1 mM PMSF) and broken by glass beads. After centrifugation the supernatant was collected (soluble fraction) and the pellet was resuspended in urea buffer (50 mM Tris pH 8.7, 150 mM NaCl, 1 mM EDTA, 1 mM protease inhibitor cocktail, 1 mM PMSF, 8 M urea). The sample was centrifuged and the supernatant was collected (urea fraction) while the pellet was resuspended in SDS buffer (SDS 10%, 1 mM protease inhibitor cocktail, 1 mM PMSF; SDS fraction).

### SDS-PAGE and western blot analysis

The samples were boiled for 3 min in the Laemmli buffer system and then were loaded on a 12% poly-acrylamide analytical SDS gel. Electrophoresis in the separating gel was conducted at 30 mA for 5 hours. After the stacking gel was removed, transfer of proteins from SDS gels to 0.45 μM Protran Nitrocellulose Transfer Membrane was done for 1 hour at 250 mA.

#### Blocking and incubation with primary antibody to detect Sam2p-GFP

The nitrocellulose paper was then incubated in 5% milk made in TBS-Tween over night at 4°C with shaking. Monoclonal anti-GFP antibody (Living Colors A.v JL-8, Diatech Labline) was diluted 1:1000 in 5% milk/TBS-Tween and applied to the nitrocellulose membrane. After incubation for 2 hours at room temperature with shaking, the membrane was washed in three changes of TBS-Tween over 25 min.

#### Blocking and incubation with primary antibody to detect actin

Monoclonal anti-actin antibody (Abcam 2Q1055) was diluted 1:1000 in 5% milk/TBS-Tween and applied to the nitrocellulose membrane. After incubation for 3 hours at room temperature with shaking, the membrane was washed in three changes of TBS-Tween over 25 min.

#### Incubation with secondary antibody and chemiluminescent detection

Rabbit anti-Mouse IgG (FC) secondary antibody, AP (alkaline phosphatase) conjugate was diluted 1:15000 in 5% milk/TBS-Tween and applied to the nitrocellulose membranes for 1 hour at room temperature with shaking. The membranes were washed in four changes of TBS-Tween or TBS over 30 min and dried. The membranes were incubated with CDP-Star Chemiluminescent Substrate for 5 min at room temperature under gentle agitation. The nitrocellulose membranes were then exposed to Pierce Cl-x posure film to reveal Sam2p-GFP and actin signals, respectively. Bands were quantified with ImageJ 1.48 software.

### Fluorescence microscopy analysis

CEN.PK 113-5D and BY4741 *SAM2*GFP strains were observed in a Nikon ECLIPSE 90i fluorescence microscope (Nikon) equipped with a 100X objective. Emission fluorescence due to GFP was detected by B-2A (EX 450–490 DM505 BA520) filter (Nikon). Digital images were acquired with a CoolSnap CCD camera (Photometrics) using MetaMorph 6.3 software (Molecular Devices).

### Flow cytometric analysis

For identification of dead or severely compromised cells, cells were washed three times (Tris-HCl 50 mM, MgCl_2_ 15 mM, pH 7.7) and resuspended in propidium iodide (PI) solution 0.23 mM. Samples were then analyzed using a CYTOMICS FC 500 flow cytometer (Beckman Coulter) equipped with a diode laser (excitation wavelength 488 nm). The fluorescence emission was measured through a 670 nm long pass filter (FL3 parameter) for PI signal. The sample flow rate during analysis did not exceed 600–700 cells/s. Threshold settings were adjusted so that the cell debris was excluded from the data acquisition; 25000 cells were measured for every sample. Data analysis was performed afterwards with Cyflogic 1.2.1 software (©Perttu Terho & ©CyFlo Ltd).

### AXP extraction and quantification

ATP, ADP and AMP were extracted and quantified as described in [50]. Briefly, extraction was performed in 0.52 M TCA containing 17 mM EDTA. After centrifugation, supernatants were neutralized with 2 M Tris-base. Neutralized samples were then analyzed by HPLC with a Zorbax Eclipse XDB-C18 LC column (150 × 4.6 mm) (Agilent Technologies) kept at 20°C. Sample elution was carried out using a mobile phase consisting of acetonitrile and tetrabutylammonium buffer (0.005 M tetrabutylammonium hydrogensulfate, 0.01 M Na2HPO4) at pH 7.0, using a flow rate of 1 mL min^-1^. A gradient was applied, where acetonitrile was increased from 6% to 25% and then back to 6%, as described in [50]. Adenonucleotides were detected with a photodiode array detector at 260 nm and peak identities were confirmed by co-elution with standards (Sigma-Aldrich). Concentrations were determined using calibration curves of standard solutions. The energy charge (E_c_) was calculated from the following equation:

$$ {\mathrm{E}}_{\mathrm{c}}\kern0.5em =\kern0.5em \left(\left[\mathrm{A}\mathrm{T}\mathrm{P}\right]\kern0.5em +\kern0.5em 0.5\kern0.5em \times \kern0.5em \left[\mathrm{A}\mathrm{D}\mathrm{P}\right]\right)/\left(\left[\mathrm{A}\mathrm{T}\mathrm{P}\right]\kern0.5em +\kern0.5em \left[\mathrm{A}\mathrm{D}\mathrm{P}\right]\kern0.5em +\kern0.5em \left[\mathrm{A}\mathrm{M}\mathrm{P}\right]\right) $$

### Extracellular metabolites and pH determination

Residual glucose and lactic acid produced were determined via high-performance liquid chromatography (HPLC, Model 1100, Agilent Technologies) using an Aminex HPX-87H ion exchange column 300 mm × 7.8 mm (Bio-Rad) thermostated at 60°C. The mobile phase was 5 mM sulphuric acid with a flow of 0.6 ml/min. Lactic acid was detected with an UV-detector at 210 nm. Glucose was detected with a RI detector, kept at 45°C.

The pH of the medium was measured with a pH-meter on fresh media or culture supernatants, after cells removal by centrifugation.

### Statistical analysis

All statistical analysis, where *p*-values are indicated, was performed using a two-tails, unpaired, heteroscedastic Student’s *t*-test.

## Additional files

Additional file 1: Figure S1. Western blot analysis of the fractions obtained from sequential protein extraction for the strain CEN.PK 113-11C *SAM2*GFP. Cells were grown in shake flasks in minimal (YNB) medium with 2% w/v glucose without or with the addition of different concentrations of lactic acid (pH 5, pH 3, 12 g/L and 20 g/L lactic acid at pH3) and then three protein sub-fractions were obtained after sequential extraction in Tris buffer (Native, **A**), 8 M urea **(B)** and 10% SDS **(C)**. The Sam2p-GFP levels were evaluated after 16 hours after inoculation using an anti-GFP antibody. Samples were normalised according to cell number. β-actin levels have been detected as control. Bands have been quantified by ImageJ 1.48 software. Histograms refer to the ratio (%) of Sam2p/Actin normalized to the values at pH 5. LA: lactic acid. Additional file 2: Table S1. Statistical evaluation of differences in lactic acid production.
