# Supplementary figures and images for: Changes in SAM2 expression affect lactic acid tolerance and lactic acid production in Saccharomyces cerevisiae
Source: Microb Cell Fact. 2014 Oct 30;13:147. doi: 10.1186/s12934-014-0147-7 (PMC4230512; doi:10.1186/s12934-014-0147-7)

## Slide 1
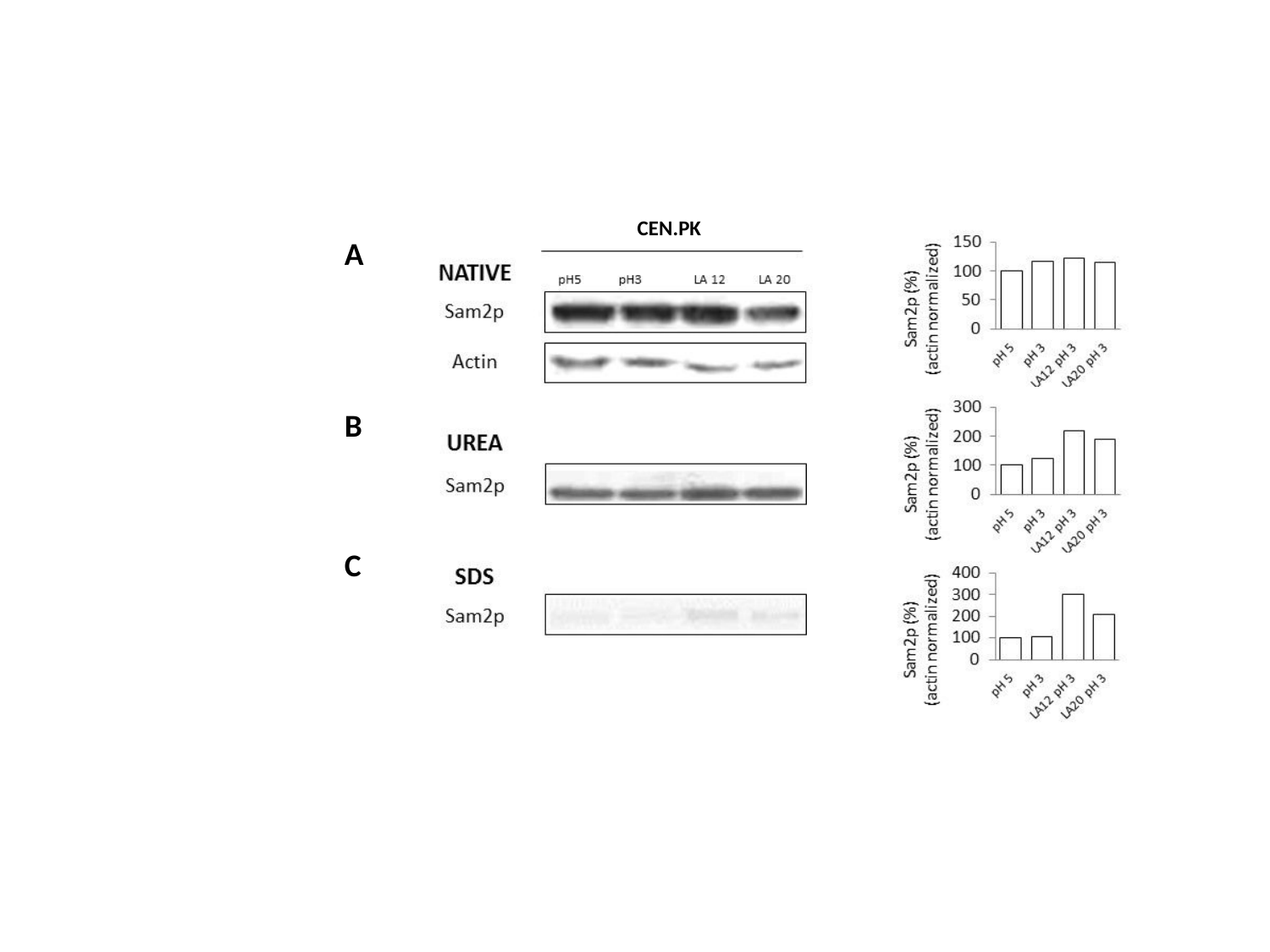

CEN.PK
A
B
C

Supplement: Additional file 1: Figure S1. — Western blot analysis of the fractions obtained from sequential protein extraction for the strain CEN.PK 113-11C SAM2GFP. Cells were grown in shake flasks in minimal (YNB) medium with 2% w/v glucose without or with the addition of different concentrations of lactic acid (pH 5, pH 3, 12 g/L and 20 g/L lactic acid at pH3) and then three protein sub-fractions were obtained after sequential extraction in Tris buffer (Native, A), 8 M urea (B) and 10% SDS (C). The Sam2p-GFP levels were evaluated after 16 hours after inoculation using an anti-GFP antibody. Samples were normalised according to cell number. β-actin levels have been detected as control. Bands have been quantified by ImageJ 1.48 software. Histograms refer to the ratio (%) of Sam2p/Actin normalized to the values at pH 5. LA: lactic acid. [file 12934_2014_147_MOESM1_ESM.ppt]
