# Supplementary material for: Changes in SAM2 expression affect lactic acid tolerance and lactic acid production in Saccharomyces cerevisiae
Source: Microb Cell Fact. 2014 Oct 30;13:147. doi: 10.1186/s12934-014-0147-7 (PMC4230512; doi:10.1186/s12934-014-0147-7)
Supplement: Additional file 2: Table S1. — Statistical evaluation of differences in lactic acid production. [file 12934_2014_147_MOESM2_ESM.docx]

**Table S1 – Statistical evaluation of differences in lactic acid production**

|  | **95% CI**^1^ **(*z* low and *z* up**^2^**, g/L)** | | | | ***t*-test *p*-values** |
| --- | --- | --- | --- | --- | --- |
| **Time (h)** | **m850** | | **m850 *sam2Δ*** | | **m850 *vs* m850 *sam2Δ*** |
| 14,5 | 12.85 | 15 | 12.72 | 16.38 | 0.5997 |
| 22 | 22.41 | 25.06 | 21.56 | 28.18 | 0.5833 |
| 38 | 45.42 | 47.8 | 47.25 | 52.18 | 0.1167 |
| 46 | 53.71 | 54.29 | 54.55 | 60.11 | 0.1423 |
| 63 | 63.17 | 63.88 | 66.04 | 67.89 | 0.0103 |
| 70 | 64.58 | 66.7 | 68.57 | 69.82 | 0.0087 |

Data for three independent experiments are reported.

^1^ Confidence Interval

^2^ Lower and upper endpoints of the CI
